# Luteolin as potential treatment for Huntington's disease: Insights from a transgenic mouse model

**DOI:** 10.1111/cns.70025

**Published:** 2024-09-03

**Authors:** Abuelnor Mohammed, Azza Ramadan, Asim Ahmed Elnour, Ali Awadallah Ali Mohamed Saeed, Nadia Al Mazrouei, Fahad T. Alsulami, Yousef Saeed Alqarni, Vineetha Menon, Abdulla Al Amoodi, Sami Fatehi Abdalla

**Affiliations:** ^1^ Department of Basic Medical Sciences College of Medicine‐Dar Al Uloom University Riyadh Saudi Arabia; ^2^ Department of Histology and Embryology, School of Basic Medical Sciences, Tongji Medical College Huazhong University of Science and Technology Wuhan China; ^3^ College of Pharmacy Al Ain University Abu Dhbai United Arab Emirates; ^4^ AAU Health and Biomedical Research Center Al Ain University Abu Dhabi United Arab Emirates; ^5^ Program of Clinical Pharmacy, College of Pharmacy Al Ain University Abu Dhabi United Arab Emirates; ^6^ Department of Pharmacology, Faculty of Clinical and Industrial Pharmacy National University, Mycetoma Research Center Khartoum Sudan; ^7^ Department of Pharmacy Practice and Pharmacotherapeutics, Faculty of Pharmacy University of Sharjah Sharjah United Arab Emirates; ^8^ Clinical Pharmacy Department, College of Pharmacy Taif university Taif Saudi Arabia; ^9^ Department of pharmacy practice, college of pharmacy Imam abdulrahman bin faisal university Dammam Saudi Arabia; ^10^ Department of Pharmacy Practice, College of Pharmacy Gulf Medical University Ajman United Arab Emirates; ^11^ Ambulatory Healthcare Services, Academic Affairs Abu Dhabi Health Services (SEHA) Abu Dhabi United Arab Emirates; ^12^ Clinical Department, College of Medicine Almaarefa University (Diriyah) Riyadh Saudi Arabia

**Keywords:** huntingtin aggregations, huntington's disease, luteolin, motor behavior, neuroprotection

## Abstract

**Aims:**

The study aimed to evaluate the potential benefits of luteolin treatment in Huntington's disease (HD), an inherited progressive neurodegenerative disorder.

**Methods:**

HD N171‐82Q transgenic and WT mice received luteolin or vehicle for treatment at 6 weeks of age. The mice's body weight changes and survival rates were monitored throughout the study, and a series of motor functional tests were conducted. Serum level of the marker NfL was also determined. Immunohistochemical staining and western blotting were utilized to assess the expression of huntingtin aggregates.

**Results:**

Luteolin treatment enhanced survival and prevented weight loss in HD mice compared to the vehicle‐treated HD group. Furthermore, the luteolin‐treated HD mice exhibited enhanced motor coordination and balance and significantly reduced motor dysfunction. Also, luteolin decreased serum NfL levels in HD mice. Notably, the accumulation of huntingtin aggregates was significantly reduced in the brain's cortex, hippocampus, and striatum of luteolin‐treated HD mice compared to the vehicle‐treated HD group.

**Conclusion:**

Luteolin holds promise as a therapeutic agent for improving survival outcomes, managing motor dysfunction, and reducing huntingtin aggregates in HD. The findings are of significance as currently, there are no approved therapeutic interventions that reverse HD pathology or slow down its progression.

## Graphical abstract

Huntington's disease (HD) is an inherited progressive neurodegenerative disorder characterized by motor dysfunction and premature mortality. Luteolin, a natural flavonoid with known antioxidant and neuroprotective properties, has shown promise in various neurodegenerative diseases. This study aimed to evaluate the potential benefits of luteolin treatment in a mouse model of (HD). To assess the effects of luteolin treatment, HD N171 82Q transgenic mice received luteolin administration, while another group received a vehicle treatment for comparison. The mice's body weight changes and survival rates were monitored throughout the study. Additionally, they underwent a series of motor function tests, including the rotarod test, balance beam test, and limb clasping test. Immunohistochemical staining and western blotting were utilized to assess the localization and expression of huntingtin aggregates, respectively. Findings revealed a significant protective effect of luteolin treatment against premature mortality and prevented weight loss in HD mice compared to the non‐luteolin‐treated group. Furthermore, the luteolin‐treated mice exhibited enhanced motor coordination and balance and significantly reduced motor dysfunction‐related manifestations, such as dystonic movements. Immunohistochemistry analysis and protein quantification demonstrated a decrease in the accumulation of huntingtin aggregates in the brain's cortex, hippocampus, and striatum of luteolin‐treated mice compared to the vehicle‐treated group. These findings collectively indicate that luteolin holds promise as a therapeutic agent for managing motor dysfunction, reducing huntingtin aggregations, and improving survival outcomes in individuals affected by Huntington's disease. The findings are of significance as currently, there are no approved therapeutic interventions that reverse HD pathology or slow down its progression.

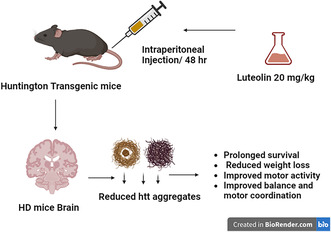

## 1 INTRODUCTION

Huntington's disease (HD) is an inherited autosomal dominant progressive neurodegenerative disorder characterized by the clinical features of psychiatric disturbances, cognitive decline, dementia, and progressive motor dysfunction that manifest as involuntary uncontrolled movement (chorea).^1^, ^2^ While HD is known to affect the central nervous system, emerging evidence suggests it is also a peripheral disease affecting multiple organs and tissues, including skeletal muscle. Weight loss was noted among HD patients at the presymptomatic stage and during disease progression. Weight loss was thought to be due to many factors, including muscle atrophy, decreased glucose metabolism, and gastrointestinal abnormalities.^3^ The onset of adult HD symptoms can occur early, averaging between 30 and 50 years old.^4^ Furthermore, according to a Norwegian study, the mean death age of HD patients was 63.9 years, in comparison to 76.9 years in the general population,^5^ indicating shorter life expectancy among HD patients.

HD is caused by the expansion of CAG repeats in the first exon of the huntingtin gene, which encodes the huntingtin (Htt) protein.^6^ Extended poly‐Q repeats cause misfolding of the N‐terminal Htt fragments, leading to abnormal protein interactions and aggregation of mutant Htt.^7^ All mutant Htt protein constructs form aggregates in the cytoplasm, but only shorter N‐terminal fragments produce nuclear aggregates.^8^, ^9^ In addition to aggregate formation, another major hallmark of HD is the degeneration of the GABA‐producing medium spiny neurons found within the brain striatum (>90% degeneration). One of the proposed mechanisms for involuntary movements in humans is a loss of neurons.^3^, ^10^ Neurodegeneration was also observed in other brain regions, evidenced by cerebral cortex atrophy, with the cortical pyramidal neurons being the most damaged, resulting in the disease associated motor symptoms. Other regions, including the cerebellum and hippocampus, were shown to be affected.^3^

It has been suggested that mutant huntingtin aggregates are toxic due to impaired vital cellular component functions.^11^, ^12^ The exact pathological mechanisms mediating the disease's onset and progression remain unclear. However, impaired ubiquitin‐proteasome system, autophagy function,^13^, ^14^ oxidative stress, and apoptosis are implicated in the disease pathogenesis mechanism.^15^, ^16^ Increased aggregation correlates with increased apoptosis.^17^

The flavonoids, which are phytochemical compounds found in vegetables, fruits, and plants, such as epigallocatechin‐3‐gallate, kaempferol, and apigenin, were shown to be neuroprotective molecules.^18^, ^19^, ^20^

Luteolin (3′,4′,5,7‐tetrahydroxyflavone), a flavonoid found mainly present in the glycosylated form, is well known for its antioxidant, antiapoptotic, anti‐inflammatory, and anticancer properties.^21^ Also, luteolin was shown to have anti‐neurodegenerative disease activity.^22^ For instance, luteolin attenuated hydrogen peroxide mediated cytotoxicity in primary cultured neurons.^23^ Furthermore, luteolin reversed amyloid protein‐induced neurotoxicity in an Alzheimer's disease (AD) cell model.^24^, ^25^ It has been reported that luteolin could penetrate the blood–brain barrier in vivo.^26^ Luteolin consumption improved spatial working memory and restored expression of inflammatory markers in the hippocampus of aged mice, indicating beneficial neuroprotection of luteolin.^18^, ^27^ Also, luteolin mediated neuroprotective effect has been well established in AD, Parkinson's disease (PD), and traumatic brain injury.^28^, ^29^ However, the role of luteolin in HD remains to be further investigated. Hence, the purpose of the current study was to investigate the putative neuroprotective effect of the flavonoid luteolin in a mutant Htt mouse model of HD. The findings will shed light on the efficacy and safety of the plant‐derived luteolin as a potential therapeutic agent for slowing down HD progression.

## 2 MATERIALS AND METHODS

### 2.1 Generation of HD transgenic mice and treatment

HD N171‐82Q‐81 transgenic mice (Jackson Laboratories, 003627) were bred and crossed to the wild type (WT) strain C57BL/6 mice for colony formation. Each experimental group had six males and five females. All animal experiments were approved by the Institutional Animal Care and Use committee of Huazhong University of Science and Technology, China.

### 2.2 Genotyping

Genotyping of mice's tail biopsies was performed using Quantitative PCR (qPCR), whereby total RNA was isolated using the RNeasy Lipid Tissue Mini Kit (Qiagen). Reverse transcription reactions the Superscript III First‐Strand Synthesis System (Invitrogen, 18080–051). Quantitative PCR analysis was performed in the using Real plex Mastercycler thermocycler (Eppendorf). Details regarding primers for genotyping and verification of CAG repeats; and qPCR conditions for amplification is included in Materials and methods S1.

### 2.3 Treatments and survival tests

HD N171‐82Q and WT mice, received an intraperitoneal (IP) injection of luteolin (20 mg/kg/every other day) treatment while the other groups of HD N171‐82Q and WT mice received vehicle treatment. Treatments were administered at 6 weeks of age till 24 weeks. For the survival studies, mice were observed until they lost 30% of their body weight or exhibited a moribund appearance (based on poor exercise performance), at which time they were euthanized or noted to have died spontaneously.

### 2.4 Motor Functions performance assessment

All behavioral tests were conducted weekly starting from 6 week of age till 24. Motor function assessment of the mice was performed using the Rotarod test with a Rotarod apparatus (Columbus Instruments) as previously described.^30^ For the limb clasping test mice were hung by the tail and observed for 60 s. Mice with normal limb extension were given a score of 0. Foreleg clutching was scored 1, and hindleg clutching was scored 2. The recorded scores and the clutch duration (time) for each group were averaged.^31^ Also, motor coordination was assessed using the balance beam test as previously described.^32^

An experimental outline depicting the study time course, experimental groups, treatments, and functional tests are found in Figure S1.

### 2.5 Blood collection and Neurofilament light chain (NFL) quantification

Blood sample was collected from the tail vein every 2 weeks according to the previous published protocol.^33^ Serum NfL concentration level was determined using the Simoa NfL assay Quanterix according to manufacturing protocol (Quanterix Corp) as previously described.^34^

### 2.6 Immunohistochemistry

For the immunohistochemical detection of Htt aggregates, brain tissue sections were incubated with the EM48 antibody (1:500) at 4 h for 35–40 h. Brain micrographs were captured in the cerebral cortex, hippocampus, and striatum brain regions using a Nikon Labophot‐2 microscope with a 40× lens. Quantification of aggregates was performed using Image‐pro Plus 6.0 software.

### 2.7 Western blot

Brain tissues proteins were extracted using NP‐40 lysis buffer protein concentrations were determined using the BCA assay (Thermo Fisher Scientific). Tissue lysate were subject to western blotting. Details regarding the above methodologies are included in Materials and Methods S1.

### 2.8 Statistical analysis

Data were presented as the mean ± SD. Statistical analyses were performed using SPSS Statistics 17.0 software. The normality of the data distribution was confirmed using Gaussian distribution. Student's t test was utilized when comparing means of two group. Multiple group comparisons for the data collected at sequential time points (for a single animal from week 6–week 20) were analyzed using two‐way repeated‐measures ANOVA, followed by Tukey's multiple comparison test. Differences were considered significant if *p* < 0.05.

## 3 RESULTS

### 3.1 Luteolin treatments prolonged the lifespan and reduced weight loss of HD N171‐82Q transgenic mice

N171‐82Q transgenic HD mice have been previously well characterized.^35^, ^36^, ^37^, ^38^ In brief, N171‐82Q transgenic HD mice have progressive phenotypes that resemble those of humans with HD where they have a shortened lifespan of about 22 weeks and develop motor disorders from the age of 12 weeks.^35^ Furthermore, these mice show weight loss, abnormal movements, and brain atrophy.^35^ Also, neuronal loss was observed in the cortex, hippocampus, striatum, cerebellum, and brainstem.^37^ This was associated with the accumulation of the Htt peptide in intracellular inclusions and neuritic aggregates.^37^ Hence, this well‐characterized HD mouse model was used to test the potential protective effects of luteolin treatments against mutant htt toxicity. The experimental groups were luteolin‐treated HD, vehicle‐treated HD, luteolin‐treated WT, and vehicle‐treated WT mice.

**FIGURE 1:**
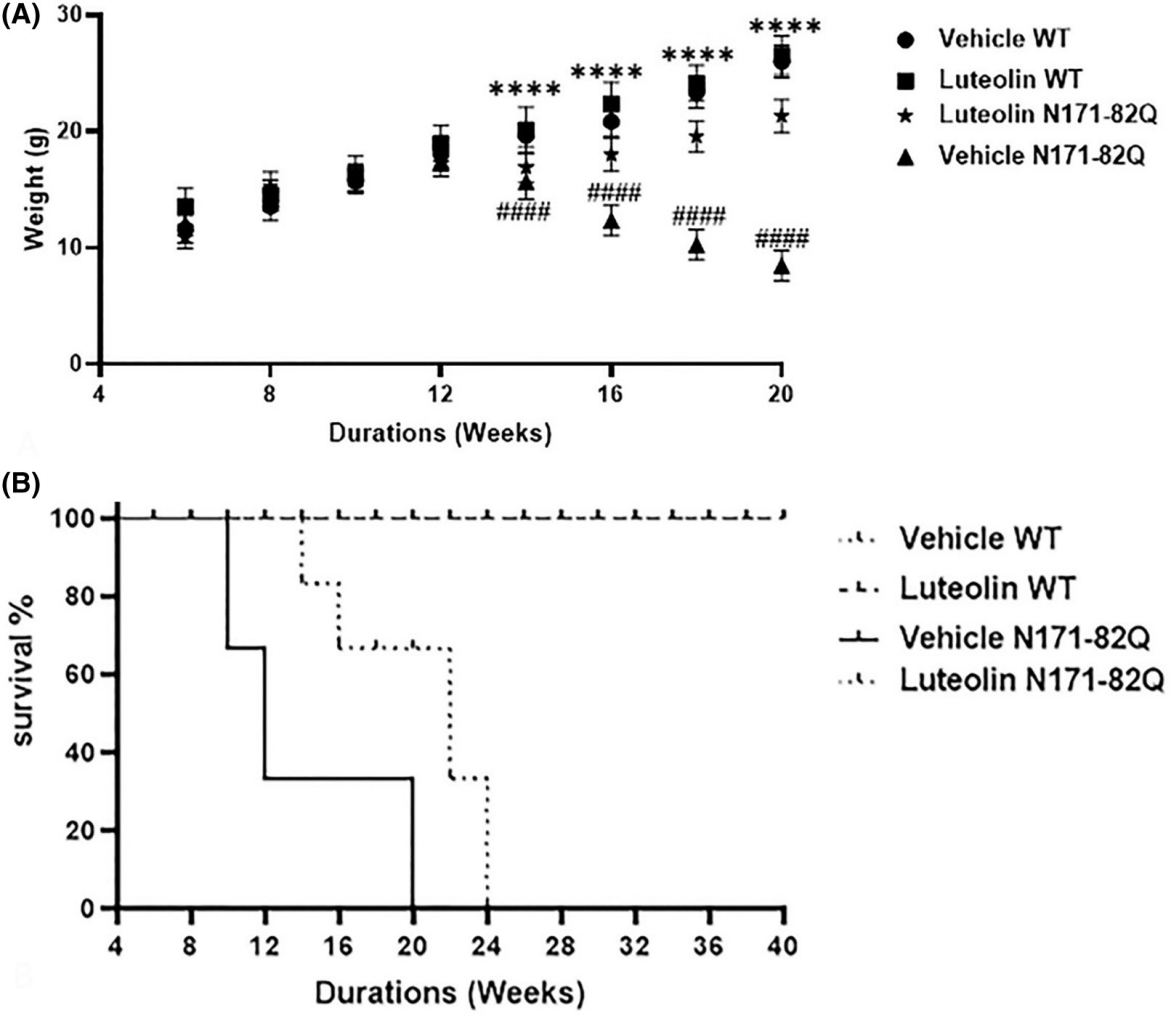
Luteolin prevented progressive body weight loss and increased the survival duration of the HD N171‐82Q mice. (A) The body weights of HD N171‐82Q and WT mice treated with vehicle or luteolin were measured weekly starting at age 6 weeks. A repeated measure two‐way ANOVA examined the effect of treatment groups and treatment duration (weeks) on weight change. There was a statistically significant interaction between the effects of treatment groups and treatment duration on weight change F (21, 279) = 42.30, *p* < 0.0001. Simple main effects analysis showed that treatment groups significantly affected weight change, F (3, 279) = 262.3, *p* < 0.0001. Similarly, treatment duration also affected weight change, F (7, 279) =163.3, *p* < 0.0001. Post hoc Tukey's analysis showed that the vehicle‐treated HD transgenic mice experienced a substantial significant decrease in body weight from week 14 and onwards compared with vehicle‐treated WT mice (^####^ *p* < 0.0001). Luteolin treatment significantly prevented the body weight loss of the HD transgenic mice compared to the vehicle‐treated HD transgenic mice (****p < 0.0001). *n* = 11, data presented as mean ± S.D. (B) The Kaplan–Meier survival curve was used to study the survival rates of HD N171‐82Q and WT mice treated with vehicle or luteolin. The vehicle‐treated HD transgenic mice died prematurely from week 10. Luteolin treatment prolonged the survival of the HD mice. WT mice receiving vehicle or luteolin continued surviving beyond the study period of 24 weeks. *n* = 11, data presented as mean ± S.D.

We initially assessed body weight loss and survival in HD mice. We commenced weighing both WT and transgenic mice once a week starting from 6 weeks of age. Over time, we observed a significant decline in body weight in the HD mice compared to the WT mice. This progressive weight loss became evident at 14 weeks of age, indicating a characteristic manifestation of Huntington's disease. To evaluate the potential benefits of luteolin treatment, we administered luteolin, 20 mg/kg/every other day to a group of HD mice, while another group of HD mice received a vehicle treatment for comparison purposes. Remarkably, the luteolin‐treated HD mice did not lose weight compared to the HD mice treated with the vehicle (Figure 1A), suggesting that luteolin mediated protective properties against the weight loss associated with HD. Furthermore, we compared the body weight of the luteolin‐treated HD and the WT mice‐treated with the vehicle or luteolin. The results revealed no significant differences between these three groups. This outcome further strengthens the notion that luteolin has the potential to mitigate the undesirable effects of HD on body weight.

To further assess the potential beneficial effect of luteolin, we conducted a survival test as shown by the Kaplan–Meier curve, where we closely monitored the occurrence of animal deaths over the experiment period of 24 weeks. Our findings revealed that the HD transgenic group receiving the vehicle treatment experienced premature mortality, with the first deaths occurring at 10 weeks of age. By the time the mice reached 20 weeks of age, a staggering 100% (all) of the vehicle‐treated HD transgenic mice had expired (Figure 1B). The mean age of death for this group was calculated to be 18.6 ± 2.3 weeks. However, in contrast to the vehicle HD‐treated group, the mice treated with luteolin displayed a significant protective effect against premature death. Luteolin treatment effectively prolonged the lifespan of the transgenic HD mice by 25.5% when compared to the vehicle HD control group (Figure 1B). This finding highlights luteolin's role in extending lifespan and improving survival outcomes in HD. It is noteworthy to mention none of the WT mice that received luteolin or vehicle treatment died within the study time frame of 24 weeks. In fact, these mice continued to live for up to 40 weeks. This observation further supports the notion that luteolin treatment specifically targets and confers its protective effects on the HD transgenic mice, without posing any adverse effects on the survival of normal, WT animals (Figure 1).

### 3.2 Luteolin treatment improved HD mice's motor‐associated functions

**FIGURE 2:**
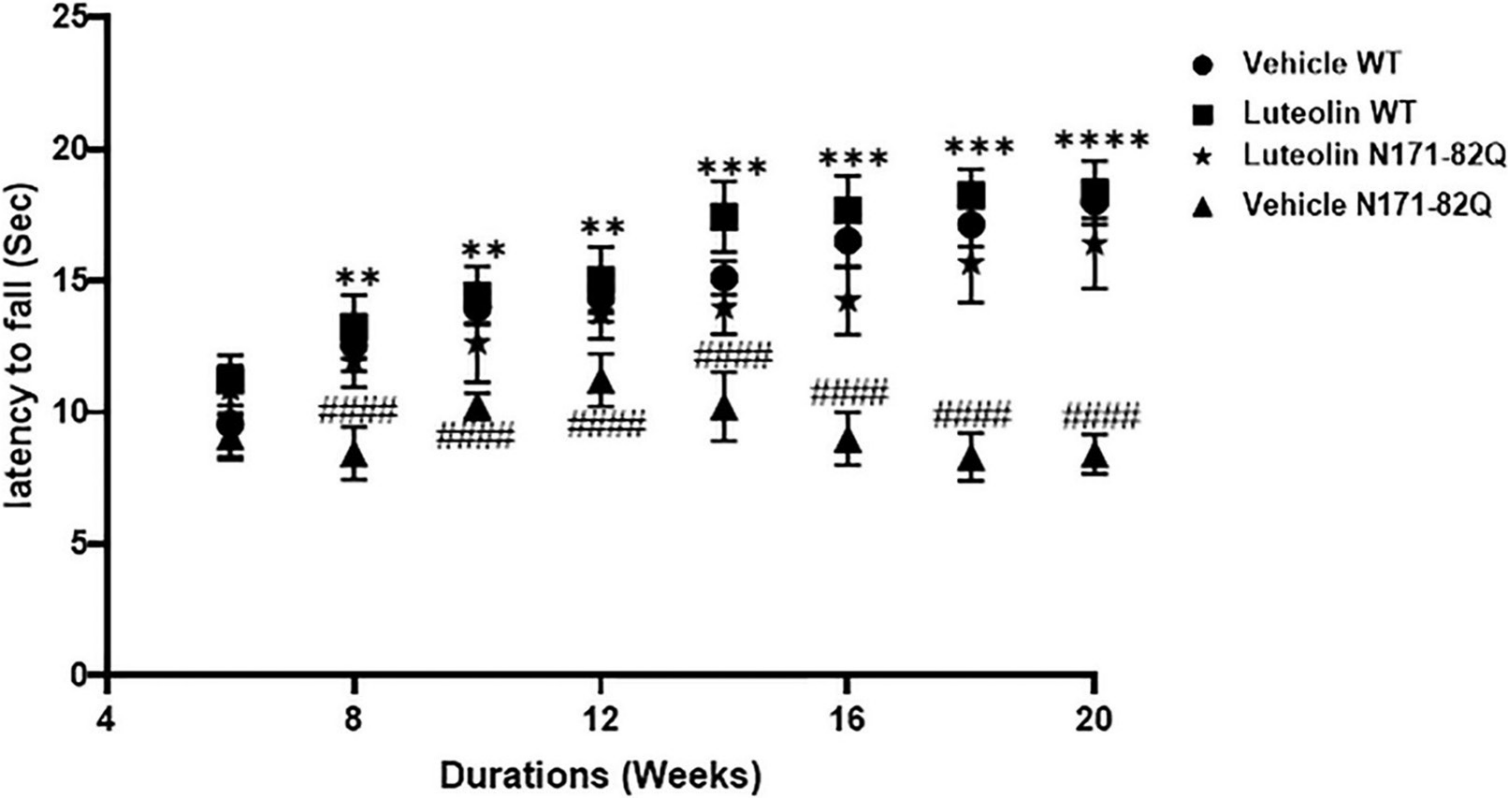
Luteolin increased mean rotarod latency to fall duration in the HD transgenic mice. The rotarod test was conducted on the experimental groups starting at 6 weeks of age. The mice were placed onto a rotating rod that accelerated from 4 to 40 rpm over 15 s and then held at 40 rpm for 60 s. The latency to fall was recorded as an average from three trials on the rod every week. Two‐way ANOVA repeated measures showed a statistically significant interaction between treatment groups X treatment duration (weeks), F (21, 279) = 15.24, *p* < 0.0001. Main effects analysis showed treatment groups significantly affected latency to fall, F (3, 279) = 417.8, *p* < 0.0001. Furthermore, treatment duration significantly affected the latency to fall, F (7, 279) = 101.8, *p* < 0.0001. Post‐Tukey's test analysis: Vehicle‐treated HD transgenic mice showed a significant decrease in latency to fall compared to the vehicle‐treated WT mice, from week 8 and onwards, ^####^ *p* < 0.0001. In contrast, luteolin‐treated HD transgenic mice exhibited significantly increased latency time and improved motor performance compared to the vehicle‐treated HD transgenic mice, **p < 0.01, ***p < 0.001, ****p < 0.0001. *n* = 11, data presented as mean ± S.D.

We conducted a series of behavioral tests, including the rotarod, balance beam, and limb clasping tests, to assess motor performance and disease progression in HD N171‐82Q transgenic mice after luteolin treatment. The rotarod acceleration test was used to assess motor performance functions. The rotarod trial was started at 6 weeks of age once a week. The vehicle‐treated HD (control) group showed a decrease in motor performance over time evident by the short duration of latency to fall compared to the WT groups that received vehicle (*p* < 0.0001) or luteolin (Figure 2). The HD group that received luteolin treatment showed a marked improvement in motor performance over time as latency to fall was longer compared to the vehicle HD‐treated group starting as early as week 8 (*p* < 0.001, Figure 2). In fact, no significant difference in rotarod performance was observed between the luteolin HD and the WT groups. We observed no decline in performance in HD mice treated with luteolin, rather the motor performance of the treated transgenic mice was close to that of WT mice treated with vehicle or luteolin.

**FIGURE 3:**
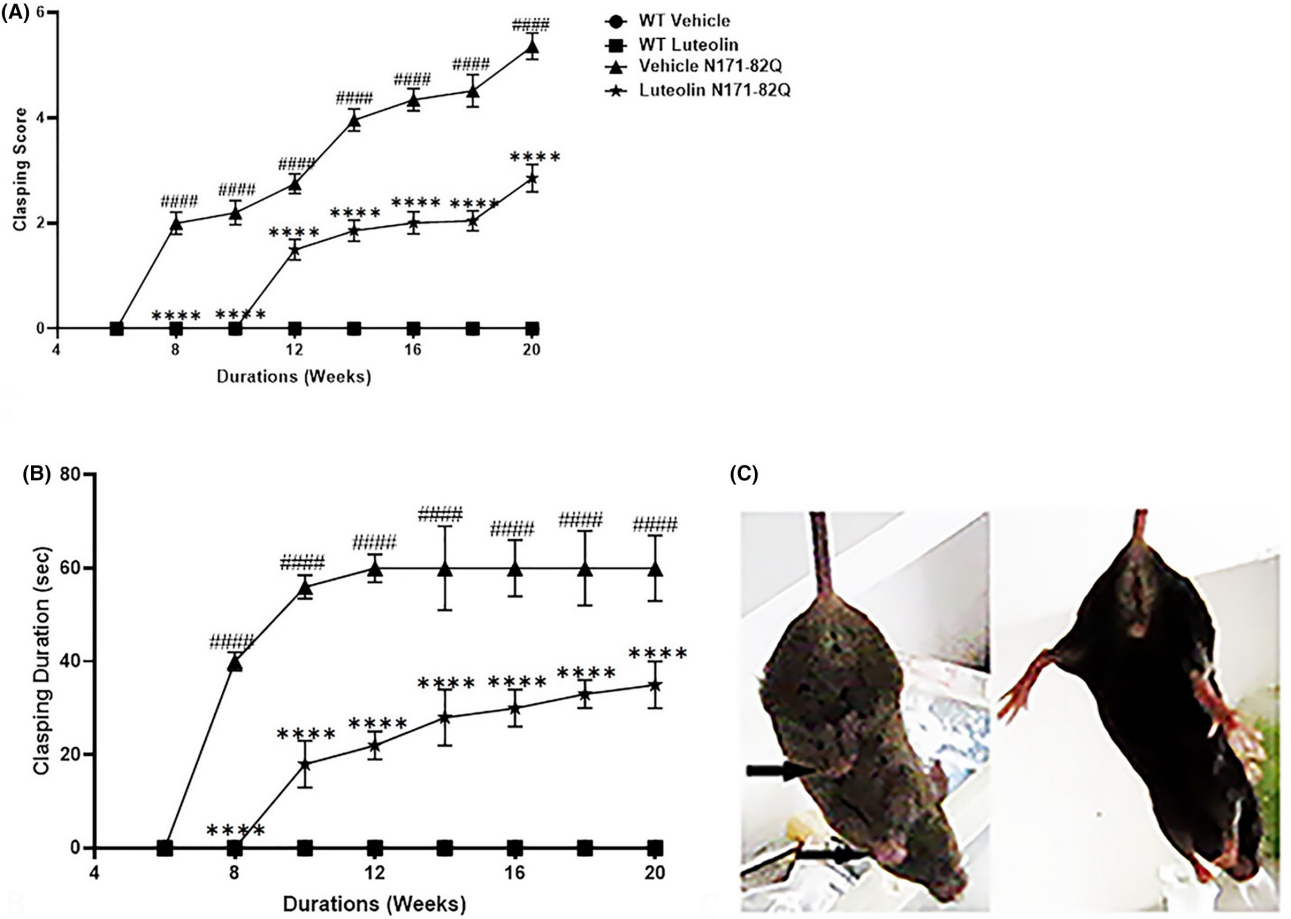
Luteolin ameliorated limb clasping in HD transgenic N171‐82Q mice. HD transgenic and WT mice treated with vehicle or luteolin underwent limb clasping test starting at 6 weeks of age. (A) Represents limb clasping scores test. Two‐way ANOVA repeated‐measures analysis showed that there was a significant effect of treatment groups x duration (F (21, 279) = 525.0, *p* < 0.0001), treatment (F (3, 279) = 10,441, *p* < 0.0001), and duration (F (7, 279) = 1357, *p* < 0.0001) on clasping scores. Post hoc Tukey's analysis revealed that HD vehicle‐treated transgenic mice showed notable hindlimbs and forelimbs clasping, which progressed over time compared to non‐limb clasping vehicle‐treated WT mice (^####^ *p* < 0.0001). Luteolin injections in the HD transgenic mice significantly reduced clasping scores compared to the vehicle‐treated HD transgenic mice, indicating improved motor performance (Tukey's post hoc test, ****p < 0.0001). (B) Represents the duration of the limb clasping during the test. Two‐way ANOVA revealed significant treatments' or duration's effect on clasping durations (F (3, 279) = 4480, *p* < 0.0001) and (F (7, 279) = 357.0, *p* < 0.0001), respectively. Significant interaction between treatment x treatment duration on clasping durations was also noted (F (21, 279) = 156.1, *p* < 0.0001). Luteolin treatment in HD transgenic mice significantly reduced the clasping duration compared to vehicle‐treated HD mice (Tukey's post hoc test, ****p < 0.0001). (C) Representative images of HD transgenic mice, 20 weeks of age, demonstrate limbs clasping in HD mice treated with the vehicle (denoted by black arrows). In contrast, the luteolin‐treated mice showed normal limbs lateral extensions. WT vehicle data points (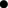) are overlapping with WT luteolin (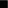). *n* = 11, data presented as mean ± S.D.

Regarding assessing motor impairment, we employed a limb clasping test. This limb clasping phenomenon became evident at 8 weeks of age in HD vehicle‐treated mice and it intensified as the disease progressed (Figure 3). In contrast, the luteolin‐treated transgenic mice displayed a distinct improvement in motor function. Most of these mice exhibited clasping of only the forelimbs, evident by the low clasping scores and duration (Figure 3A,B) or no clasping at all. This observation suggests that luteolin treatment exerted a protective effect on motor function, mitigating the formation of dystonic postures and improving motor response. In WT mice, no limb clasping was observed, indicating normal motor function. However, in the HD transgenic control mice, we noted the presence of dystonic postures in the forelimb and the hindlimbs, which led to self‐clasping behavior. (Figure 3C). These findings provide further evidence of the beneficial impact of luteolin in ameliorating motor dysfunction in HD mice.

**FIGURE 4:**
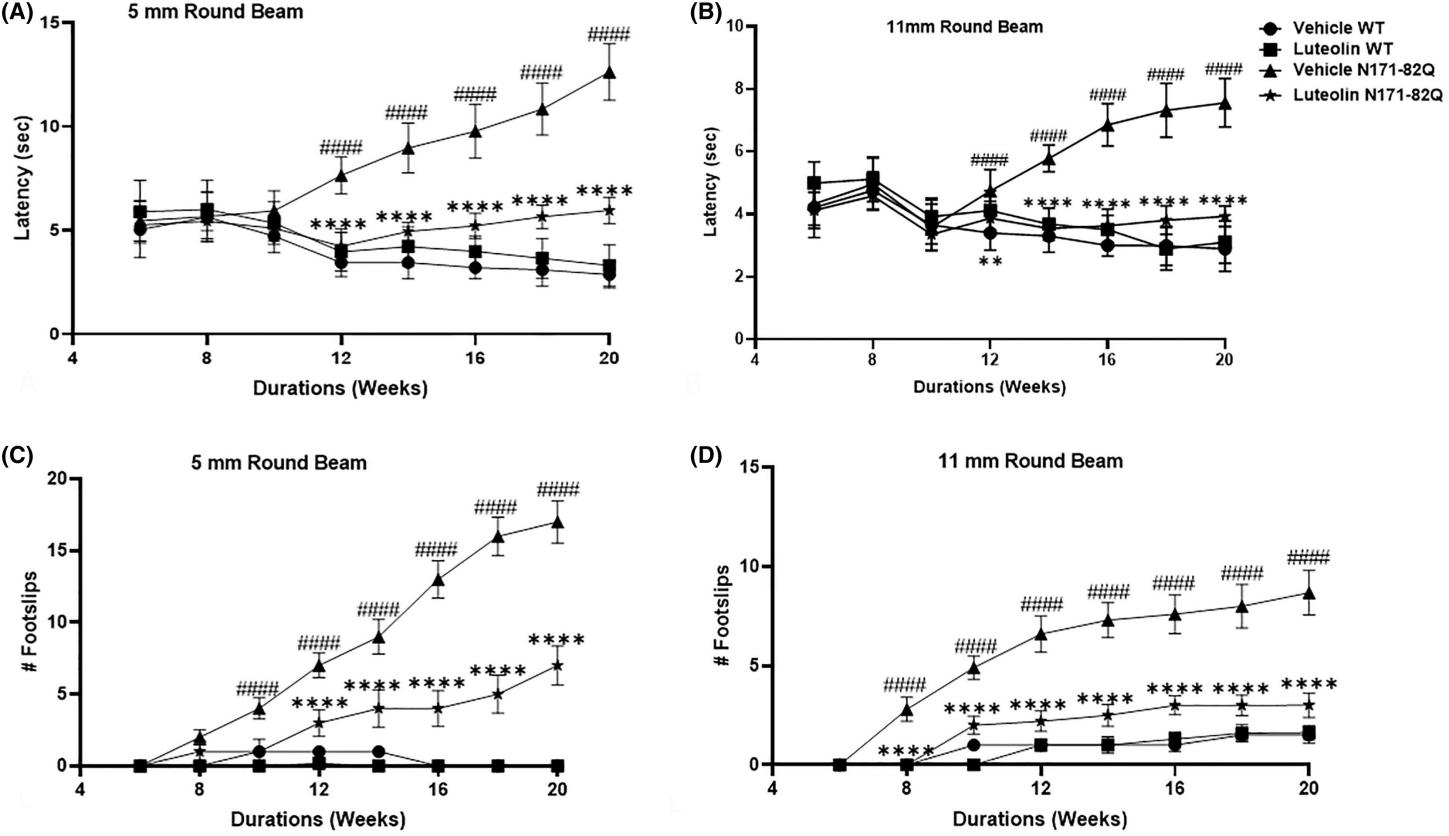
Luteolin improved the balance and motor coordination of the HD transgenic mice in the beam walking test. The balance beam test was performed on HD transgenic and WT mice treated with vehicle or luteolin weekly at 6 weeks of age. (A) Latency to walk across a raised 5 mm beam; two‐way repeated‐measures ANOVA with Tukey's post hoc test: Treatment group × treatment duration interaction F (21, 279) = 25.95, *p* < 0.0001, treatment group effect F (3, 279) = 270.1, *p* < 0.0001, and treatment duration effect F (7, 279) = 6.328, *p* < 0.0001. Tukey's post hoc: Latency to walk across the 5 mm beam declined progressively in vehicle‐treated HD mice starting at week 12 versus WT mice (^####^ *p* < 0.0001). However, luteolin administration significantly enhanced the latency of walking in vehicle‐treated HD mice (****p < 0.0001). (B) The latency to walk to cross an 11 mm round beam; two‐way repeated‐measures ANOVA with Tukey's post hoc test: Treatment group × duration interaction F (21, 279) = 19.62, *p* < 0.0001, treatment group effect F (3, 279) = 145.5, *p* < 0.0001, and duration effect F (7, 279) = 13.47 *p* < 0.0001. Tukey's post hoc: Latency to cross the round beam declined in vehicle HD mice at week 12 (vs vehicle WT mice, ^####^ *p* < 0.0001); however, it was improved upon luteolin treatment at week 14 and onwards (vehicle HD vs. luteolin HD, **p = 0.0043, ****p < 0.0001). (C) The average frequency of foot slips on the 5 mm beam; two‐way repeated‐measures ANOVA with Tukey's post hoc test: Treatment group × duration interaction F (21, 279) = 180.7, *p* < 0.0001; treatment group effect F (3, 279) = 2355, *p* < 0.0001 and duration (weeks) effect F (7, 279) = 409.1, *p* < 0.0001. Tukey's post hoc: The average frequency of foot slips that significantly increased progressively in vehicle‐treated HD (vs. vehicle WT mice, ^####^ *p* < 0.0001) was improved via the administration of luteolin (vs. vehicle HD mice, ****p < 0.0001). (D) The average frequency of foot slips on the 11 mm round beam; two‐way repeated‐measures ANOVA with Tukey's post hoc test: Treatment group × weeks interaction F (21, 279) = 63.87, *p* < 0.0001, treatment group effect F (3, 279) = 1690, *p* < 0.0001, and duration treatment (weeks) effect F (7, 279) = 362.9, *p* < 0.0001. Tukey's post hoc: The significant progressive increased frequency of foot slips in vehicle HD mice (vs. vehicle WT mice, *p* < 0.0001) was markedly reduced in luteolin HD mice (vs. vehicle HD mice, ****p < 0.0001) as early as week 8. *n* = 11, data presented as mean ± S.D.

To evaluate the fine motor coordination and balance capabilities of HD mice, we utilized the beam‐walking test. The results demonstrated that control vehicle‐treated HD mice faced significant challenges when traversing both the wider 11 mm beam and the narrow 5 mm beam, starting at ~12 weeks of age. Their latency to traverse the beam increased with age compared to the control WT mice, indicating difficulties in motor coordination and balance (Figure 4A,B). Additionally, these HD transgenic mice exhibited a significantly higher frequency of foot slips on both beams, with the frequency increasing as they aged (Figure 4C,D). In contrast, luteolin treatment for the transgenic mice led to an improvement in motor coordination. The luteolin‐treated mice displayed a significant decrease in latency time when traversing both beams and a lower frequency of foot slips over time in comparison to non‐luteolin HD mice recipient (*p* < 0.0001, Figure 4). These results clearly demonstrate the positive impact of luteolin treatment on motor function and balance in HD mice. Furthermore, we observed similar trends in the beam‐walking test when evaluating luteolin‐treated HD mice and the WT groups. These data highlight the positive influence of luteolin treatment in enhancing motor coordination and balance capabilities in HD mice.

### 3.3 Luteolin decreased serum level of neurofilament light chain (NfL) in HD mice

**FIGURE 5:**
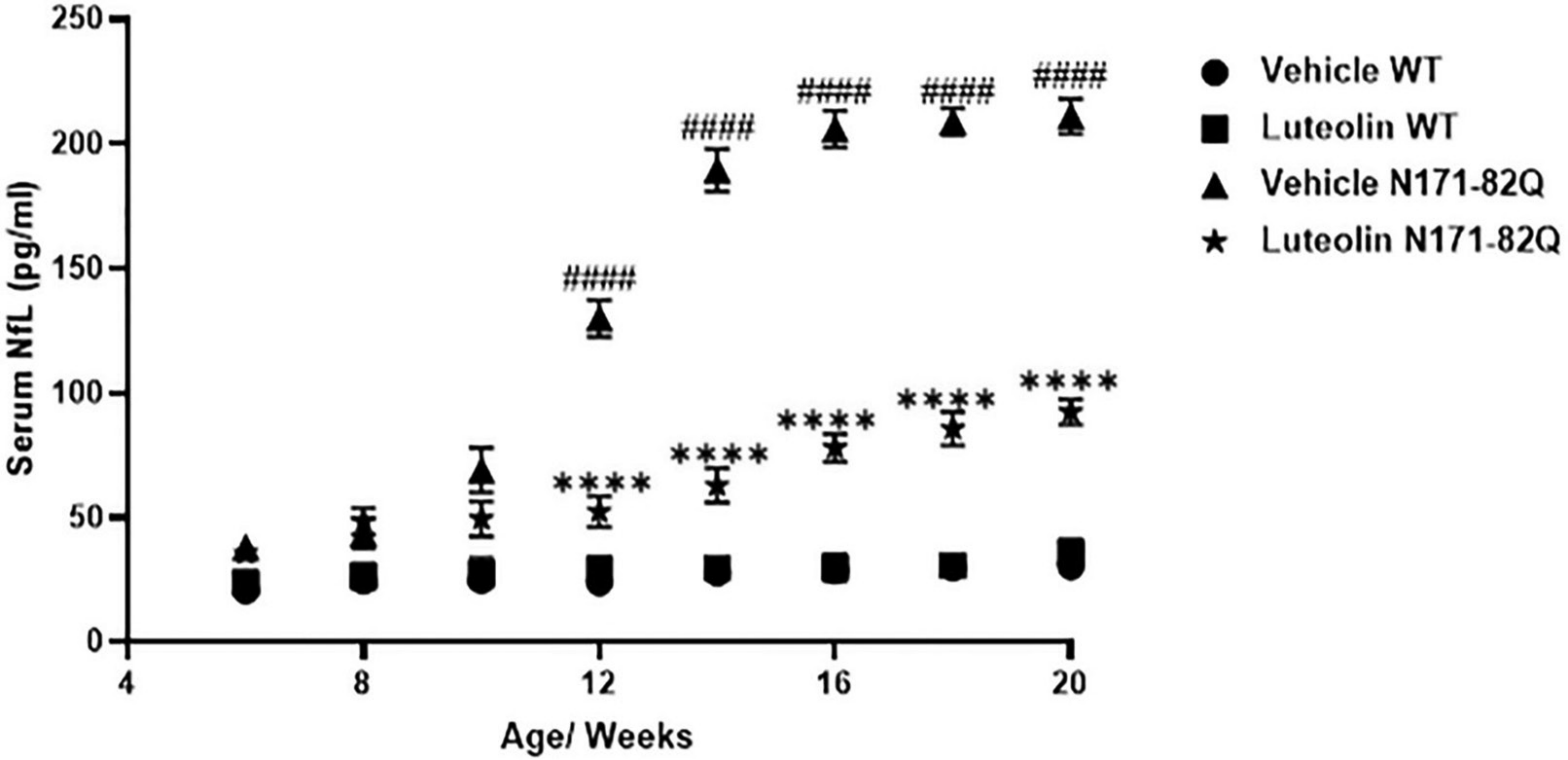
Luteolin decreased the neurodegenerative biomarker neurofilament light chain (NfL). Serum levels in the HD N171‐82Q transgenic mice. The serum NfL was measured every 2 weeks starting from the 6th week of age. During the 14 weeks of luteolin administration, two‐way ANOVA repeated‐measures analysis revealed treatment groups x treatment duration interaction affected significantly NfL serum levels, F (21, 279) = 399.7, *p* < 0.0001. The treatment group effect was F (3, 279) = 6499, *p* < 0.0001, while the treatment duration effect was F (7, 279) = 938.9, *p* < 0.0001. NfL levels that were significantly elevated in the vehicle HD group starting from week 12 (vs. vehicle WT group, ^####^ *p* < 0.0001) were decreased upon luteolin administration (vs. vehicle HD mice, ****p < 0.0001) (post hoc analysis via by Tukey's test). WT vehicle data points (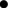) are overlapping with WT luteolin (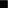). *n* = 11, data presented as mean ± S.D.

Cerebrospinal fluid and blood NfL has been characterized as biomarker for neurological disorders.^39^, ^40^ Our study tracked serum NfL levels in the four experimental groups, starting from 6 weeks of age, every 2 weeks. Over time, vehicle‐treated HD mice displayed a significant rise in serum NfL levels compared to vehicle or luteolin‐treated WT mice (*p* < 0.0001, Figure 5) which could be indicative of ongoing neuronal degeneration in the HD mice. In contrast, HD mice treated with luteolin showed a drastic decrease in serum NfL levels (*p* < 0.0001, Figure 5) compared to vehicle‐treated HD mice. Notably, both vehicle and luteolin‐treated WT mice had the lowest serum NfL concentrations in the study.

### 3.4 Luteolin treatment reduced mutant Htt aggregation in the brain of N171‐82Q transgenic mice

**FIGURE 6:**
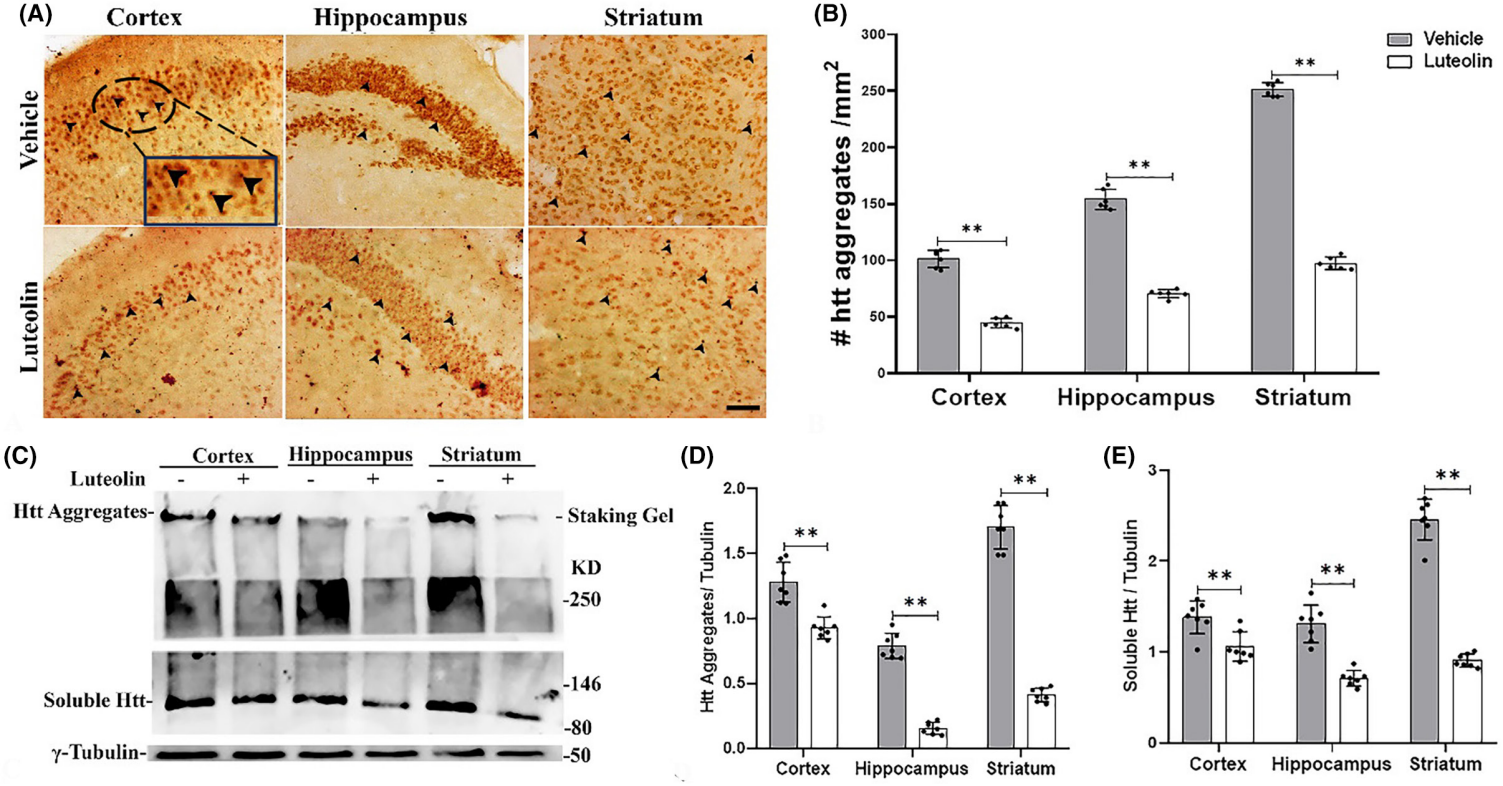
Luteolin reduced the formation of the htt aggregates in the brain of HD N171‐82Q transgenic mice. (A) Immunohistochemistry detection of the Htt aggregates (denoted by black arrowheads) in the brain tissue of HD transgenic mice treated with vehicle or luteolin. Brain micrographs were captured in the cerebral cortex, hippocampus, and striatum brain regions using a Nikon Labophot‐2 microscope with a 40× lens (Scale bar: 50 μm). (B) Quantifications of the Htt aggregates represented on the micrographs of the HD transgenic mice's cerebral cortex, hippocampus, and striatum treated with vehicle or luteolin. The entire field of view of the individual representative immunohistochemistry images shown above was used for quantification. Luteolin treatment of HD transgenic mice significantly reduced Htt aggregates compared to vehicle‐treated HD mice. *n* = 6, data represented as mean ± S.D., **p < 0.01 versus vehicle‐treated HD mice. (C) Western blot detection of the aggregated and soluble htt protein in the HD mice's brain's cortex, hippocampus, and striatum treated with vehicle or luteolin. Tubulin was used as a loading control. Densitometry analysis of western blots shows that luteolin treatments significantly decreased the expression of the aggregates (D) and soluble (E) htt protein in the HD transgenic mice brain's cortex, hippocampus, and striatum. *n* = 7, data represented as mean ± S.D., **p < 0.01 versus vehicle‐treated HD mice (Student's *t*‐test).

The mutated Htt forms abnormal intracellular aggregates in the neurons in different parts of the brain of HD mice.^37^ These aggregates have been linked to neuronal dysfunction and premature death, and it has been proposed to form a spectrum of oligomeric species and different huntingtin oligomers in vitro that exhibit varying degrees of cytotoxicity.^41^, ^42^ To investigate the impact of luteolin treatment on the accumulation of mutant huntingtin (Htt) in the brains of HD mice, we performed immunohistochemical staining for visualization of Htt expression and western blotting to detect aggregated and soluble Htt exon 1 fragments in the various brain regions. Our immunostaining analysis revealed a high density of detectable positive Htt aggregates in the cortex, hippocampus, and striatum of vehicle‐treated HD mice (Figure 6A,B). However, when examining the brains of HD mice treated with luteolin, a significant reduction in the number of Htt aggregates was observed compared to the vehicle‐treated group (Figure 6A,B). This substantial decrease in the number of aggregates confirmed the efficacy of luteolin treatment in diminishing the development and formation of neuronal inclusions in HD transgenic mice.

Furthermore, the results were confirmed by western blot analysis. The analysis of brain samples lysates from HD mice revealed a substantial accumulation of soluble and aggregated mutant Htt protein in the cortex, hippocampus, and striatum (Figure 6C–E). However, luteolin treatment significantly altered the formation of these mutant Htt inclusions, leading to a dramatic inhibition and prevention of aggregate and soluble Htt protein accumulation in the brain regions of HD mice (Figure 6C–E). Overall, the significant decrease in the mutant Htt protein aggregates number, that is, accumulation as well as the reduction of aggregated and soluble mutant Htt expression, highlight the potential therapeutic role of luteolin in mitigating the pathological effects associated with HD.

## 4 DISCUSSION

Our study revealed that the administration of luteolin to the HD mouse model, prevented weight loss, prolonged survival, and improved motor performance. Remarkably, the luteolin normalized motor performance to an extent that is similar to non‐diseased WT mice. Furthermore, luteolin reduced the histopathological hallmark of HD that is Htt aggregation.

The process of aging seems to accelerate in HD patients, thought to be due to the exacerbation of Htt mediated toxicity.^43^ Indeed, our findings show that HD mice died prematurely. However, luteolin treatment extended the life span of the HD mice, suggesting its essential role in longevity. In non‐diseased *Caenorhabditis elegans* and *D. melanogaster* females, luteolin treatment extended lifespan.^44^ This suggests luteolin may help to extend lifespan in HD as well as in non‐diseased models. Also, luteolin reduced H_2_O_2_ mediated senescence in (HEI‐OC1) cells derived from immortalized mouse cochlea cell line.^45^

In addition to the reduction of life span, the HD mice were noted to lose weight progressively as they aged. Histological analysis of muscle tissue obtained from HD patients and HD mouse model showed granular deposits/inclusions that were positive for Huntingtin staining.^46^, ^47^, ^48^ Also, it was shown that the increase in the granular/inclusion size and number aligned with muscle atrophy formation. However, whether muscle atrophy is due to neuronal degeneration, or an intrinsic muscle defect is still unclear. It has been suggested that Huntington's deposit accumulation mediated impairment of the protein quality control system could contribute to muscle pathology.^49^ Hence, the HD mice's weight reduction could have resulted from muscle loss. However, luteolin‐treated HD mice displayed similar weights to non‐diseased WT mice. Indeed, multiple lines of evidence have demonstrated the protective effect of luteolin against muscle wasting.^50^

At the functional level, we showed that luteolin significantly delayed the onset and the severity of motor dysfunctions in HD mice. This was evident by the improvement of balance and motor coordination in luteolin‐treated HD mice relative to non‐treated mice. Furthermore, it was observed that luteolin‐treated HD mice displayed limited forearm dystonia or no dystonia at all. Similarly, luteolin administration improved exploration in the triple transgenic (3 × Tg‐AD) diseased mice, evident by increase in frequency of grid crossing, which indicate improved locomotion.^51^ Oxidative stress is a key pathological process implicated in neurodegenerative diseases including AD, PD, and HD. It was shown that oral or intrastriatal luteolin administration in sodium nitroprusside‐induced oxidative stress mouse model, conferred protection against brain damage and motor dysfunction.^52^ Also, in the transgenic Drosophila PD model, administration of luteolin improved the flies climbing and reduced neuronal oxidative stress dose‐dependently.^53^ CDKL5 deficient mouse model is a neurodevelopmental disease model characterized by many deficiencies, including motor stereotyping. Luteolin administration to CDKL5 +/− mice improved locomotion and reduced hindlimb clasping compared to vehicle‐treated, which indicates improvement of in motor stereotyping.^54^

NfL, a neuronal cytoplasmic protein, is a major component of the neuronal cytoskeleton and is believed to function primarily to provide structural support for the axon and to regulate axon diameter. NfL get released, in large quantities, following axonal damage or neuronal degeneration.^39^ A progressive elevation of serum NfL levels in HD 171‐82Q mice was detected as the disease progressed in our study which suggests ongoing neuronal damage or degeneration. Note that degenerated neurons with apoptotic features have been previously reported in HD 171‐82Q mice.^55^ In agreement, an increase in NfL levels was reported in HD R6/2 transgenic mice.^56^ Also, Soylu‐Kucharz et al.^56^ (2017) demonstrated a correlation between NfL increased levels in cerebrospinal fluid and serum, and neurodegeneration along with disease severity in the R6/2 transgenic mice. We showed that luteolin remarkably decreased the NfL levels in the HD 171‐82Q mice. The findings imply that luteolin preserves neuronal structural integrity by preventing its damage or degeneration.

Gross examination of the HD N171‐82Q transgenic brain tissue in the current study showed that luteolin reduced Htt aggregates in the cortical, hippocampus and striatal regions. Furthermore, luteolin decreased the expression of mutant Htt aggregates and soluble forms in those regions. The aggregation of mutant htt protein into monomers, oligomers, or inclusion bodies, plays a crucial role in the pathogenesis of HD. Oligomeric aggregates are small soluble and can be found in the nucleus or cytoplasm. Inclusion bodies are insoluble proteins rich in packed mutant proteins that form rod‐shaped aggregates called fibrils. They are also found in the cytoplasm and nucleus. Both oligomers and inclusion bodies cause neuronal cell dysfunction as they sequester vital proteins that are important in various cellular processes, such as transcription, translation, cytoskeletal mitochondrial function, the protein quality control system, and vesicle transport. Inclusion bodies can also impair ER function. The monomeric form can spread to other cells via the lysosomal secretory pathway and cell‐to‐cell tubular junctions, resulting in aggregate formation in neighboring cells and causing further dysfunction in other brain regions.^57^

Other neurodegenerative diseases are also associated with insoluble fibril‐rich aggregates, such as aggregates of *α*‐synuclein protein in PD, tau and amyloid proteins in AD, and prion proteins in prion diseases.^58^ Concurrent with our findings, luteolin decreased the deposition of β‐amyloid in the hippocampus of the AD 3 × Tg‐AD mice.^51^ Also, luteolin reduced the expression of beta‐amyloid and beta‐amyloid cleaving enzyme‐1, essential for amyloid‐beta formation, in the brains of Aβ1–42‐, AD mice. Furthermore, luteolin improved synaptic function in the hippocampus region.^59^ In streptozotocin‐induced AD rat model, luteolin increased the hippocampus CA1 pyramidal layer thickness in comparison to the control group.^60^ Similar effects were observed in the PD cell model as luteolin decreased the expression of α‐synuclein in arsenic‐treated dopaminergic PC12 cells.^61^

### 4.1 Limitations

The study demonstrated the pronounced effect of luteolin‐mediated life extension and prevention of motor‐related dysfunction in HD mice. However, the study has some limitations. The putative gender specific contribution (male vs. females) in luteolin‐mediated beneficial effects in HD was not investigated.

### 4.2 Future directions

Further studies are needed to elucidate the underlying mechanisms by which luteolin exerts its protective effects in HD. Additionally, investigations into optimal dosage, treatment duration, and long‐term effects are needed to fully understand the potential of luteolin as a therapeutic intervention for HD. Studies investigating the cause of weight loss in N171‐82Q mice is warranted. Furthermore, an investigation into the cause of the weight gain observed in luteolin‐treated HD mice compared to vehicle‐treated HD mice is necessary. In particular, the putative role of luteolin in preventing muscle wasting and energy expenditure.

## 5 CONCLUSION

In conclusion, luteolin treatment shows promising effects in ameliorating motor dysfunction, improving motor coordination and balance, and reducing the accumulation of mutant Htt aggregates in HD mice. These findings contribute to the growing body of evidence supporting luteolin as a promising candidate for managing the motor‐related symptoms associated with HD. Overall, the results imply that luteolin can potentially be therapeutic in HD, a condition where currently there is no treatment to cure or slow the course of disease progression.

## CONFLICT OF INTEREST STATEMENT

The authors declare that they have no known competing financial interests or personal relationships that could have appeared to influence the work reported in this paper.

## CONSENT FOR PUBLICATION

We declare our consent for the publication of our article.

## Supporting information


Materials and Methods S1.

Figure S1.

Figure S2.

## Data Availability

The data that support the findings of this study are provided upon reasonable request.
