# Supplementary material for: Luteolin as potential treatment for Huntington's disease: Insights from a transgenic mouse model
Source: CNS Neurosci Ther. 2024 Sep 3;30(9):e70025. doi: 10.1111/cns.70025 (PMC11371662; doi:10.1111/cns.70025)
Supplement: Supplementary file 1 — Materials and Methods S1. [file CNS-30-e70025-s003.docx]

**Material and Methods**

**Generation of HD transgenic mice and animal housing**

*B6C3*-*Tg*(*HD82Gln*)*81Dbo*/*J*. 003627. Common name: HD N171-82Q-81 transgenic mice (Jackson Laboratories) were bred and crossed to the wild type (WT) strain C57BL/6 mice for colony formation. The characterization of the HD N171-82Q-81 transgenic mice has been well described previously.^1-4^ Mice were housed in cages under humidity and temperature controlled conditions (12hr light:12hr dark cycle) with free access to food and water. Male and female mice were evenly distributed to the experimental groups (i.e. each experimental group had 6 males and 5 females). All animal experiments were conducted after consultation with the local animal welfare committee and approved by the Institutional Animal Care and Use committee of Tongji Medical College, Huazhong University of Science and Technology, China.

**Genotyping**

Genotyping was performed using qPCR whereby the total RNA was isolated from the mice tail biopsies using the RNeasy Lipid Tissue Mini Kit (Qiagen). Reverse transcription reactions were performed with 1.5 μg of total RNA using the Superscript III First-Strand Synthesis System (Invitrogen, 18080–051). cDNA (100 ng) was combined with 10 μl SYBR Select Master Mix (Applied Biosystems, 4472908) and 1 μl of each primer in a 20-μl reaction. The reaction was performed in the Eppendorf, Real plex Mastercycler thermocycler.

Primers for genotyping CAG repeats in transgenic N171-82Q mice were as follows: sense S26: 5’-CTA CGA GTC CCT CAA GTC CTT CCAGC-3’, antisense A151: 5’-GAC GCA GCA GCG GCT GTG CCT G-3’.^5-7^ Each PCR amplification was carried out in the following PCR conditions: 94℃ for 3min; 30 cycles of 94℃ for 30 s, 55℃ for 40 s, and 72℃ for 50 s; followed by 72 ℃ for 5 min. Using these protocols cDNA amplification was within its linear range. After amplification, the products were separated in a 2% agarose gel containing 0.03% ethidium bromide and photos of the amplified genes were taken for visualization. To verify CAG repeats stability, it was ensured that the target amplicon size of the qPCR product was obtained as a single band at the predicted size of 240 bp.

**Treatments and survival tests**

The experimental groups in our study, HD N171-82Q and WT mice, received an intraperitoneal (IP) injection of luteolin (20 mg/kg/every other day, in a volume of 5 ml/kg dissolved in DMSO) while the other groups of HD N171-82Q and WT mice received DMSO as a vehicle for treatment. Luteolin and vehicle treatment were administered from 6 weeks of age and injected at the same time every 48 hours up to 24 weeks of age. Examination of the effects of luteolin treatment on HD mice was compared to vehicle (DMSO) treated HD mice that were used as a control. In addition, to eliminate possible causes of death other than those due to the presence of the Htt transgene, we used WT, which also received DMSO injection. Also, to rule out the possible toxicity of the administration of luteolin-applied doses, the luteolin recipient WT mice group was used in this study. For the survival studies, mice were observed until they lost 30% of their body weight or exhibited a moribund appearance (based on poor exercise performance), at which time they were euthanized or noted to have died spontaneously.

**Motor Functions performance assessment**

To minimize potential environmental influences and accurately evaluate the neurological signs of HD mice, we housed them under indistinguishable conditions as the control mice and abstained from providing any special care that might mask their symptoms. Behavioral testing was conducted in a dedicated environment designed to limit external noise and visual distractions. The tests commenced at week 6 of age till week 24, every week.

**Rotarod test**

Motor function assessment of the mice was performed using the Rotarod test with a Rotarod apparatus (Columbus Instruments, Columbus, OH) as previously described.^8^ Mice performed the rotarod test weekly from 6 weeks of age. The test was performed by placing mice on a horizontally rotating rod, which was low enough to prevent animal injury due to falling but high enough to induce the fall. The rotarod accelerated from 4 to 40 rpm over 15 s and then held at 40 rpm for 90 seconds.  The trial commenced with the start of acceleration and ended when the mice fell. Three trial measurements on the rod for their latency to fall were recorded in seconds. A maximum latency of 60 seconds was defined for mice that did not fall. The mice were allowed at least 15 minutes to recover between each trial.

**Limb-clasping test**

The limb clasping test has been extensively used to recognize HD mice's neurological impairment. Once a week from 6 weeks of age and onwards, mice were hung by the tail and observed for 60 seconds. Mice with normal limb extension were given a score of 0. Foreleg clutching was scored 1, and hindleg clutching was scored 2. The recorded scores and the clutch duration (time) for each group were averaged.^9^

**Balance beam test**

Motor coordination was assessed using the balance beam test. The test apparatus consisted of a beam supported by poles. A box was placed at the end of the beam, which is considered the finish point of the test. The test takes a couple of consecutive days, starting with a couple of training and one test day. A 17mm round beam, 11mm round beam, and 5 mm square beam were used in the study. The mice were trained to traverse (cross) the beam each week to the enclosed box. The mice training progressed from the widest beam to the narrowest one, i.e. on the 17 mm round beam for the first day, the 11 mm round beam for the second day, and the 5 mm square beam for the third day, with two trails per day. Once a stable performance baseline was obtained, the mice were tested weekly in two consecutive trials on the 11 mm round beam followed by the 5 mm square beam. The latency to traverse the middle section (80 cm in length) of each beam and the number of times the hindfeet slipped off each beam were recorded for each trial. For each measurement, the mean scores of the two trials for each beam were used in the analysis.^10^

**Blood collection and Neurofilament light chain (NFL) quantification**

Blood sample was collected from the tail vein every two weeks according to the previous published protocol.^11^ The blood samples were stored at room temperature for 30 minutes, and then centrifuged at ~2500 × g for 15 minutes. Following the centrifugation supernatant (serum) was collected. blood serum samples were frozen in dry ice and stored at −80 °C until further use. Serum NfL concentration level was determined using the Simoa NfL assay Quanterix according to manufacturing protocol (Quanterix Corp,USA) as previously described.^12^

**Tissue Preparation**

Mice were anaesthetized by intraperitoneal injection of sodium pentobarbital (100 mg/kg body weight) and then perfused via the ascending aorta with 100 ml of 0.01 M sodium phosphate buffered saline (PBS) 0.85% (pH 7.4), followed by 300 ml of 0.1 M sodium phosphate buffer (pH 7.4) containing 4% paraformaldehyde. After perfusion, the brains were removed and post-fixed in the same fixative at 4°C for 4 to 6 hours. Then the brains were soaked in cold 0.1 M phosphate buffer containing 30% sucrose at 4°C overnight. The following day, tissues were quick frozen in liquid nitrogen to protect from dehydration and then cut into 30 mm pieces (ribbons) on a cryostat. Sections were collected in PBS and kept at 4°C until use.

**Immunohistochemistry**

The immunohistochemical detection of Htt aggregates was performed using the avidin-biotin-peroxidase (ABC) method. To reduce endogenous peroxidase activity and prevent non-specific antibody binding, sections were treated in 1% hydrogen peroxide-PBS for 2 hours and then in PBS containing 5% normal goat serum for 30 minutes after incubation in PBS containing 1% Triton X-100. Thereafter, the sections were incubated with the EM48 antibody (1:500) at 4 h for 35 to 40 hours, followed by incubation with biotinylated goat anti-rabbit immunoglobulin G (IgG) (1:200) at room temperature for 2 hours followed by avidin-biotin complex (1:200) at room temperature for 2 hours. Antibody and the avidin-biotin complex were diluted in PBS containing 3% Triton X-100 and 5% normal goat serum. Tissues were rinsed in PBS between incubations. Finally, the immunoreactive products of Htt aggregates were visualized by incubation with 0.02% diaminobenzidine (Sigma-Aldrich) and 0.3% hydrogen peroxide in 0.05 M Tris-HCl buffer for 10-15 minutes. The sections were then mounted on gelatin- and chromium-coated slides. Brain micrographs were captured in the cerebral cortex, hippocampus, and striatum brain regions using a Nikon Labophot-2 microscope with a 40× lens. Quantification of aggregates in the brain sections was performed using Image-pro Plus 6.0 software. All huntingtin aggregates in the captured images were counted and analyzed. The aggregates were categorized as intranuclear or neuropil aggregates. Neuropil aggregates are localized outside the cell body and their size is usually small, whereas nuclear aggregates (NIs) appeared as single inclusions within the nucleus.

**Western blot**

Brain tissues were stored in NP40 buffer (50mM Tris pH 7.4, 50mM NaCl, 0.1% Triton X-100, 1% NP40, protease inhibitor, Cocktail Pierce 78430) and 1 ml PMSF (Sigma P-7626) based on the homogenized protocol previously described (Lin et al., 2016). The lysates were sonicated and the protein concentrations were determined using the BCA assay. An equal amount of protein (80 ng/40 µl/lane) was separated on SDS-PAGE 12% gels. Proteins were then transferred to nitrocellulose membrane (GE Healthcare Life Sciences, Marlborough, MA, USA). The membrane was blocked in 5% non-fat dry milk in PBS for 30 minutes and then incubated with the primary antibodies Htt anti-mouse (EM48 antibody, Sigma-Aldrich, Cat# MAB5374, RRID: AB_2314368), 1:1000 in 3% BSA/PBS overnight at 4℃. Mouse tubulin (Sigma-Aldrich, Cat# T9026, RRID: AB_477593),1:10000 was used as a loading control. After incubation, the blots were washed, and secondary HRP-conjugated antibodies (Jackson Immuno-Research, RRID: AB_10015289) were added in 5% milk for 2 hours and then visualized using SuperSignal ECL (Pierce). The results were analyzed using a Bio-Rad Imaging Densitometer.

**References**

1. Ferrante RJ. Mouse models of Huntington's disease and methodological considerations for therapeutic trials. *Biochim Biophys Acta*. Jun 2009;1792(6):506-20. doi:10.1016/j.bbadis.2009.04.001

2. Mughal MR, Baharani A, Chigurupati S, et al. Electroconvulsive shock ameliorates disease processes and extends survival in huntingtin mutant mice. *Hum Mol Genet*. Feb 15 2011;20(4):659-69. doi:10.1093/hmg/ddq512

3. Schilling G, Becher MW, Sharp AH, et al. Intranuclear inclusions and neuritic aggregates in transgenic mice expressing a mutant N-terminal fragment of huntingtin. *Hum Mol Genet*. Mar 1999;8(3):397-407. doi:10.1093/hmg/8.3.397

4. Schilling G, Klevytska A, Tebbenkamp AT, et al. Characterization of huntingtin pathologic fragments in human Huntington disease, transgenic mice, and cell models. *J Neuropathol Exp Neurol*. Apr 2007;66(4):313-20. doi:10.1097/nen.0b013e318040b2c8

5. Wu LL, Fan Y, Li S, Li XJ, Zhou XF. Huntingtin-associated protein-1 interacts with pro-brain-derived neurotrophic factor and mediates its transport and release. *J Biol Chem*. Feb 19 2010;285(8):5614-23. doi:10.1074/jbc.M109.073197

6. Putkhao K, Kocerha J, Cho IK, Yang J, Parnpai R, Chan AW. Pathogenic cellular phenotypes are germline transmissible in a transgenic primate model of Huntington's disease. *Stem Cells Dev*. Apr 15 2013;22(8):1198-205. doi:10.1089/scd.2012.0469

7. Cho IK, Clever F, Hong G, Chan AWS. CAG Repeat Instability in the Peripheral and Central Nervous System of Transgenic Huntington's Disease Monkeys. *Biomedicines*. Aug 2 2022;10(8)doi:10.3390/biomedicines10081863

8. Deacon RM. Measuring motor coordination in mice. *J Vis Exp*. May 29 2013;(75):e2609. doi:10.3791/2609

9. Miedel CJ, Patton JM, Miedel AN, Miedel ES, Levenson JM. Assessment of Spontaneous Alternation, Novel Object Recognition and Limb Clasping in Transgenic Mouse Models of Amyloid-beta and Tau Neuropathology. *J Vis Exp*. May 28 2017;(123)doi:10.3791/55523

10. Luong TN, Carlisle HJ, Southwell A, Patterson PH. Assessment of motor balance and coordination in mice using the balance beam. *J Vis Exp*. Mar 10 2011;(49)doi:10.3791/2376

11. Parasuraman S, Raveendran R, Kesavan R. Blood sample collection in small laboratory animals. *J Pharmacol Pharmacother*. Jul 2010;1(2):87-93. doi:10.4103/0976-500X.72350

12. Sano T, Masuda Y, Yasuno H, Shinozawa T, Watanabe T. Plasma neurofilament light chain as a potential biomarker of neurodegeneration in murine brain. *Toxicol Res (Camb)*. Oct 2023;12(5):751-755. doi:10.1093/toxres/tfad063
