# Supplementary material for: Luteolin as potential treatment for Huntington's disease: Insights from a transgenic mouse model
Source: CNS Neurosci Ther. 2024 Sep 3;30(9):e70025. doi: 10.1111/cns.70025 (PMC11371662; doi:10.1111/cns.70025)
Supplement: Supplementary file 2 — Figure S1. [file CNS-30-e70025-s001.docx]

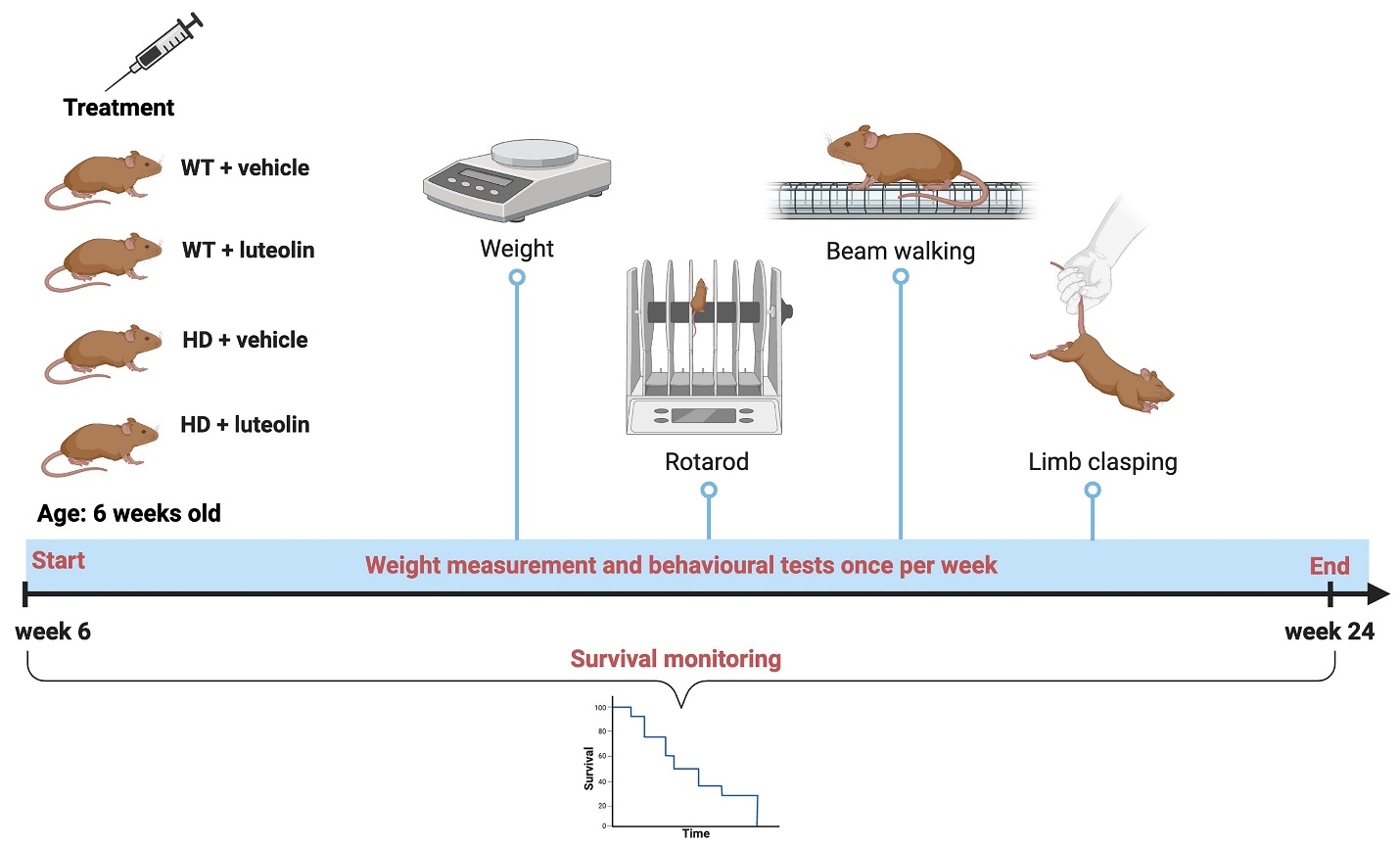


**Figure S1. Experimental outline depicting time course, experimental groups, treatments and functional tests conducted in the study.** WT and 171-82 Q (HD) mice received luteolin or vehicle treatment at the age of 6 weeks. The mice continued to receive these treatments every other day till week 24. The weight measurements and behavioural tests were conducted weekly from week 6 to week 24. During this time, the mice were monitored for survival. The experimental overview was created with BioRender.com, accessed on 21 May 2024 (Agreement number RM276QQLOR).
