# Supplementary figures and images for: Luteolin as potential treatment for Huntington's disease: Insights from a transgenic mouse model
Source: CNS Neurosci Ther. 2024 Sep 3;30(9):e70025. doi: 10.1111/cns.70025 (PMC11371662; doi:10.1111/cns.70025)

Full unedited gel/blot for Figure 6C

Figure S2

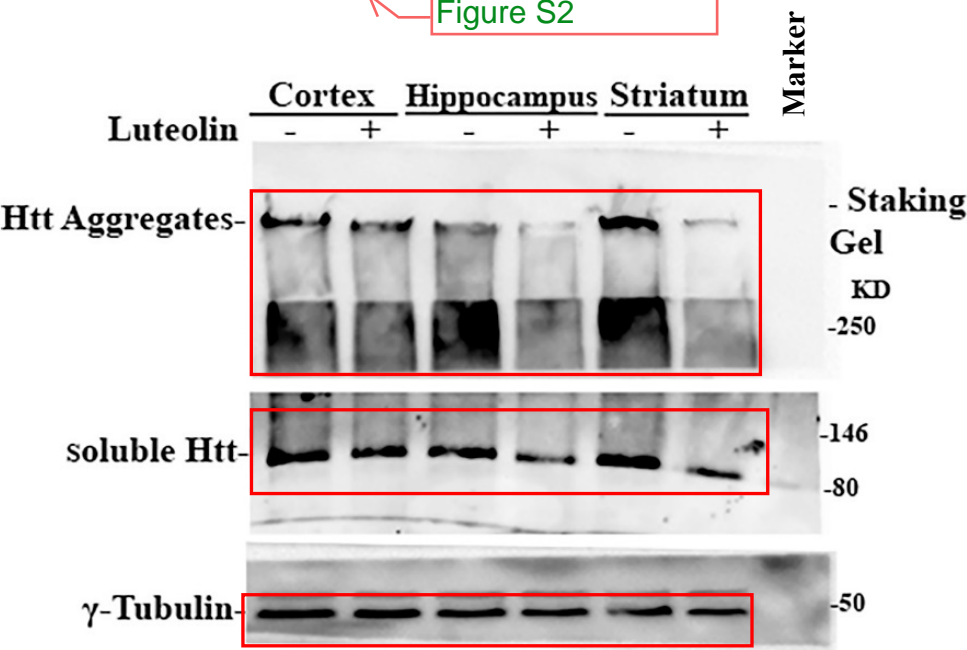

Supplement: Supplementary file 3 — Figure S2. [file CNS-30-e70025-s002.pdf]
